# The Effects of Psychopharmacologic and Therapeutic Approaches on Neuro-imaging in Obsessive-compulsive Disorder

**DOI:** 10.2174/157015913804999414

**Published:** 2013-01

**Authors:** Murad Atmaca

**Affiliations:** Firat University School of Medicine Department of Psychiatry, Elazig/Turkey

**Keywords:** OCD, Structurel, MRI, MRS, fMRI.

## Abstract

The neurobiological etiopathogenesis of OCD is still obscure. Neuroimaging studies have been very influential in shaping neurobiological models of OCD. Investigations performed within last twenty years have revealed some important findings and proposed that specific cortico-striato-thalamic circuits are involved in the mediation of its symptoms. The effects of antiobsessional drugs and cognitive behavioral therapy on structural and functional imaging have been evaluated in limited size of investigations. In structural investigations, in summary, it was found key brain regions in the pathophysiology of OCD and amygdala to change volumetrically by treatment. In functional and neurochemical investigations, by using different treatment modalities, cortico-striatal function disablements and NAA changes in a variety of brain regions were reported. In this paper, these limited data are reviewed. It is clear that there is so many things to be performed in the future researches on the effects of therapy on brains of the patients with OCD.

## INTRODUCTION

Obsessive–compulsive disorder (OCD) is an anxiety disorder characterized by the existence of obsessions and compulsions. Obsessions are recurrent, persistent intrusive unwanted thoughts, ideas, or images that produce uneasiness, apprehension, fear, or worry while compulsions are characterized by repetitive behaviors and thoughts aimed at reducing the associated anxiety, intrusive unwanted thoughts, ideas, or images. These symptoms are frequently time-consuming, and often lead to severe emotional and financial distress. The neurobiological etiopathogenesis of OCD is still obscure. Neuroimaging studies have been very influential in shaping neurobiological models of OCD. Investigations performed within last twenty years have revealed some important findings and proposed that specific cortico-striato-thalamic circuits are involved in the mediation of its symptoms [1-4]. In frontal cortex, anterior cingulate, caudate nucleus and thalamus, metabolic changes increasing during symptom provocation were demonstrated in functional imaging studies [2-4]. However, there have been inconsistencies in structural neuroimaging studies of OCD, regarding the support for this neuroanatomical model. It seems that the most important reasons for these inconsistencies are methodological differences between studies and small sample sizes. The regions aforementioned involved network in OCD are primarily innervated by serotonergic and dopaminergic neurons. In this context, the most established treatment option for OCD patients is serotonin reuptake inhibitors (SRI), alone or in combination with other medications. 40 to 60 percent of OCD patients responses to any SRI, and cognitive behavior therapy [5, 6]. On the other hand, a considerable part of the patients which may reach up to the rate of 30% to 40% does not response to available treatment modalities [5-8]. Refractoriness to treatment in OCD includes a decrease less than 35% on the Yale-Brown Obsessive-Compulsive Scale (Y-BOCS) total score at final evaluation as compared to baseline or a final score of >16 on the Y-BOCS and being no better than "minimally improved" on the Clinical Global Impression improvement item.

The effects of antiobsessional drugs and cognitive behavioral therapy on structural and functional imaging have been evaluated in limited size of investigations. In this paper, these limited data are reviewed.

## STRUCTURAL INVESTIGATIONS

In fact, the thought that some brain areas may be important in the occurrence and pathogenesis of OCD has been mentioned for he beginning of the twentieth century [1-3]. In that period, in patients demonstrating sequela of encephalitis lethargica after influenza epidemies, the observation that involuntary movements and obsessions and compulsions occurred simultaneously led the investigators to consider that basal ganglia could be causing to OCD [9]. Within the quarter of century, structural neuroimaging studies have revealed important findings which contribute us to understand the pathogenesis of OCD though neuro-biological theories of OCD are largely based on the results of the functional neuroimaging studies. As a consequence of these investigations, results have implicated the pathology of basal ganglia and frontal regions [9]. Among these regions, some areas have been determined as “key brain regions”, orbito-frontal cortex (OFC), thalamus, anterior cingulate cortex and caudate nucleus which were also emphasized these structures in the pathophysiology of OCD in a recent meta-analysis [10]. On the other hand, recently, Radua and Mataix-Cols [1] took twelve data-sets comprising 401 people with OCD and 376 healthy controls met inclusion criteria and found that OCD patients had larger regional grey matter volumes in bilateral lenticular nuclei, extending to the caudate nuclei and decreased volumes in bilateral dorsal medial frontal/anterior cingulate gyri. In their meta-ananlysis on brain volume changes in OCD, Rotge *et al*. suggested structural alteration of the thalamocortical pathways might be associated with the functional abnormalities of frontosubcortical circuits seen in OCD [10].

In an investigation on thalamus, one of key brain regions in OCD neuroanatomy, Gilbert *et al*. [11] measured neuroanatomical changes in the thalamus of patients with psychotropic drug-naive children, with OCD near the onset of illness, and before and after treatment, comparatively healthy comparison subjects and found that thalamic volumes were significantly greater in treatment-naive patients with OCD than in controls but reduced significantly after paroxetine monotherapy to levels comparable with those of controls, in correlated with the reduction in OCD symptom severity. Consequently, they suggested that OC symptom reduction by paroxetine treatment might be paralleled by a reduction in thalamic volume as much this might be specific to paroxetine treatment and could be due to other treatment options. Most recently, Hoexter *et al*. [12] performed an investigation to determine the effects of pharmacological and cognitive-behavioral therapy (CBT) on regional brain volumes in OCD patients suffered from structural magnetic resonance imaging scan before and after a 12-week randomized clinical trial with either fluoxetine or group CBT. They detected that OCD patients without any treatment had smaller gray matter volumes of left putamen, bilateral medial orbitofrontal, and left anterior cingulate cortices compared to those of healthy control subjects whereas after treatment with either fluoxetine or CBT, grey matter volumes of the left putamen of the patients were comparable to those of healthy subjects, without any significant volume changes in CBT treated patients, suggesting that fluoxetine and CBT might have different neurobiological mechanisms of action in OCD patients. Lazaro *et al*. [13] evaluated brain changes initially and after treatment and clinical improvement of 6 months' follow-up in 15 children and adolescent with OCD patients and determined that at baseline scanning, the patient group with OCD showed significantly less gray matter volume bilaterally in right and left parietal lobes and right parietal white matter while at follow up period of six months, they had comparable gray matter volumes to those of healthy control subjects, in correlation with the clinical improvement. They commented that children and adolescents with untreated OCD had gray and white matter decreases in lateral parietal cortices, improved after treatment period. 

Apart from key brain regions, the amygdala is believed to be highly relevant to the pathophysiology of OCD because on one side it has an important role in fear conditioning and on the other side it has an important target of the serotonin reuptake inhibitors (SRIs), the pharmacotherapy of choice for OCD. In this context, Szezsko *et al*. [14] found that the patients with OCD demonstrated significant asymmetry of the amygdala (L>R) before pharmacologic intervention in contrast to healthy comparison subjects. In their study, after treatment period, left amygdala volume decreased significantly in patients group, and additionally, this volume reduction in left amygdala volume was found to be correlated significantly with higher paroxetine dosage at the time of the follow-up imaging, while no significant changes in either right or left amygdala volume were evident among healthy comparison subjects from the baseline to the follow-up scan and suggested that suggest that abnormal asymmetry of the amygdala might play a role in the pathogenesis of OCD and that paroxetine treatment might be associated with a reduction in amygdala volume. In another study, it was tested whether orbitofrontal cortical volume would be reduced after the administration of anterior cingulotomy for OCD patients [15]. However, no significant volumetric reductions were found in the orbitofrontal cortex before and after anterior cingulotomy and they proposed a model of cingulo-striatal perturbation as a putative mechanism for the efficacy of anterior cingulotomy. 

## FUNCTIONAL AND NEUROCHEMICAL INVESTIGATIONS

In general, functional magnetic resonance imaging (fMRI) investigations of OCD have revealed a relationship between altered activity in the frontal-striatal areas including the OFC, dorsolateral prefrontal cortex (DLPFC), basal ganglia, and the parietal cortex, and cognitive inflexibility [16, 17]. In their review dealing with integrating evidence from neuroimaging and neuropsychological studies of OCD, Menzies *et al*. [18] emphasized that current available evidence for the orbitofronto-striatal model might not be sufficient to account for the brain basis of OCD, to detect subtle orbitofrontal-associated cognitive dysfunction in OCD patients, some of the cognitive tasks might have lacked the required sensitivity and specificity. On the other hand, during reversal learning in OCD patients and their clinically unaffected relatives, it was identified abnormally decreased activation of several cortical regions, including the lateral orbitofrontal cortex, revealing an underlying endophenotype for OCD [19].

An important fMRI investigation come from Lazaro *et al* [20] who investigated possible regional brain dysfunction in premotor cortico-striatal activity in pediatric and adolescent OCD patients and correlation brain activation with severity of obsessive–compulsive symptomatology, and possible changes in brain activity after pharmacological treatment. Both patients and controls exhibited cerebral activation involving the fronto-parietal cortex and basal ganglia while OCD patients showed significantly higher brain activation bilaterally in the middle frontal gyrus compared to healthy subjects after complex motor condition. They also determined that activation in the left insula and left putamen significantly decreased after 6 months of pharmacological treatment. Jang *et al*. [21] investigated regional N-acetylaspartate (NAA) values and changes after 12 weeks of treatment with citalopram in drug-naive OCD patients by using proton magnetic resonance spectroscopic imaging (^1^H-MRSI) and found OCD patients to have significantly decreased NAA values in the prefrontal cortex, frontal white matter, and anterior cingulate at baseline compared to those of healthy comparison subjects, with obvious increases in NAA values in the prefrontal cortex and frontal white matter in OCD patients after 12 weeks of citalopram treatment, suggesting that OCD patients were defectice in regard to neuronal viability of frontal region of the brain which might be reversible by treatment. Han *et al*. [22] reported that OCD patients exhibited considerably less activation in the dorsal and ventral frontal-striatal circuit and parietal regions under the task-switch minus task-repeat condition compared with controls at baseline while the neural responses in the ventral frontal-striatal circuit in OCD were partially normalized, with an activation deficit in dorsal frontoparietal regions after treatment, commenting the role of altered brain activation in ventral frontal-striatal regions on the pathophysiology of OCD. In Nabeyama *et al*.’ study [23], similar design was used but it was examined the alterations of brain function in OCD patients and changes after clinical improvement associated with behavior therapy, to reveal .thepathophysiology of OCD without the confounding effects of medication. In another investigation, Yoo *et al*. [24] investigated the white matter abnormalities of the patients with OCD who were drug-naive by using diffusion tensor-imaging and structural magnetic resonance imaging at baseline and the white matter changes in the patients after twelve weeks of citalopram treatment. They found that drug-naive OCD patients showed significant increases in fractional anisotropy (FA) in the corpus callosum, internal capsule and white matter in the area superolateral to the right caudate compared with helthy controls and that the increases in FA were mostly no longer observed in patients after 12 weeks of treatment compared to control subjects. They suggested white matter alterations to be related to the pathophysiology of OCD, with having reversibility with pharmacotherapy. In an investigation on thalamic blood flow, Kamini *et al*. [25] explored possible differential effects of OCD responders and non-responders to drug treatment on the regional cerebral blood flow (rCBF) by using 99mTc-HMPAO single photon emission computed tomography (SPECT) before and after 12 weeks of treatment. They determined that levels of rCBF decreased significantly in the left caudate nucleus and the left and right putamen in both treatment responders and non-responders while in the right thalamus it was found to be reduced in responders and suggested the thalamus to have an important role in the response to drug treatment. In their study, OCD patients exhibited reduced activation in the anterior cingulate gyrus and cerebellum compared to healthy controls at baseline, with increased activation in the cerebellum and parietal lobe, and decreases in activation in the orbitofrontal cortex, middle frontal gyrus, and temporal regions after clinical improvement of the course of OCD. Lazaro *et al*. [20] examined possible regional brain dysfunction in premotor cortico-striatal activity in the patients with OCD and possible changes in brain activity after pharmacological treatment. They found that the patients showed significantly higher brain activation bilaterally in the middle frontal gyrus at baseline while the activation in the left insula and left putamen reduced considerably after 6 months of the drug treatment. Most recentşy, O’Neill *et al*. [26] examined the metabolic effects of cognitive behavior therapy (CBT) on striatum, thalamus, and anterior cingulate cortex regions in pediatric OCD patients using by proton magnetic resonance spectroscopic imaging (1H MRSI) and determined N-acetyl-aspartate+N-acetyl-aspartyl-glutamate (tNAA) in left pregenual anterior cingulate cortex (pACC) to be 55.5% higher in patients than controls before CBT treatment whereas tNAA (15.0%) and CRE (23.9%) in left pACC reduced and CHO in right thalamus increased (10.6%) in all patients after treatment period. As for the left thalamus, tNAA, glutamate+glutamine (Glx), and myo-inositol (mI) which were lower were increased after CBT period. They concluded that the results might provide a support for the glutamatergic hypothesis of OCD. Freyer *et al*. [27] investigated ten unmedicated patients with OCD and 10 healthy controls during an event-related fMRI experiment before and after intensive CBT. They did not detect any significant differences between the groups or in group by time interactions in a whole-brain analysis corrected for multiple comparisons but found that the patients who showed greater clinical improvement, characterized by greater reductions in Yale-Brown Obsessive Compulsive Scale (YBOCS) scores, exhibited more stable activation in the pallidum. They concluded that psychotherapy might affect the brain activity in the caudate nucleus and the pallidum. Nakao *et al*. [28] examined regional changes in brain function by using fMRI during administration of cognitive and symptom provocation tasks before and after treatment in ten outpatients with OCD who were randomly allocated to receive either pharmacotherapy with fluvoxamine 200 mg/day or behavior therapy for 12 weeks. They found that activation in the regions, orbitofrontal, dorsolateral–prefrontal, and anterior cingulate cortices by symptom provocation decreased after symptom improvement while stroop task–related activation in the parietal cortex and cerebellum increased. In the clinical picture of the OCD, increased monitorization of error and conflict are established. This seems to be related to the anterior cingulate cortex (ACC). In this context, Huyser *et al*. [29] carried out an investigation by using fMRI during the performance of an interference task, the arrow version of the Flanker paradigm, before and after cognitive-behavioral treatment of 25 treatment naive pediatric OCD patients compared with age- and gender-matched healthy controls. They found that with an age dependence and just partially affected by the administratin of CBT, OCD patients demonstrated increased activation of the ACC during error responses and in bilateral insular cortex during high-conflict tasks and suggested that while insular dysfunction might be a state dependent condition, ACC functioning might be a vulnerability marker in pediatric OCD patients. In their study, Mataix-Cols *et al*. (2004) investigated the neural correlates of washing, checking, and hoarding symptom dimensions in OCD. While viewing alternating blocks of emotional and neutral pictures, and imagining scenarios related to the content of each picture type, the patients were scanned, with the main outcome measure of blood oxygenation level-dependent response. The patients exhibited significantly increased activation in putamen/globus pallidus, thalamus, and dorsal cortical areas (checking); left occipitotemporal regions (aversive, symptom-unrelated); bilateral ventromedial prefrontal regions and right caudate nucleus (washing); and left precentral gyrus and right orbitofrontal cortex (hoarding) and compared to controls. These results revealed that symptom dimensions in OCD could be mediated by different parts of of frontostriatothalamic circuits.

## CONCLUSION

The present review reveals that drug treatment and psychotherapetic approaches affects the brain both structurally and functionally in the patients with OCD. However, there is limited investigations on this issue. This limitation is also one of main limitation of our this review because beyond limited data no comparison studies were performed. It is clear that there is so many things to be performed in the future researches on the effects of therapy on brains of the patients with OCD.
